# MRI Versus Arthroscopy in the Detection of Glenoid Labral Tears of the Shoulder: A Cross-Sectional Study

**DOI:** 10.7759/cureus.108936

**Published:** 2026-05-15

**Authors:** Aditi Singh, Soumya Ranjan Nayak, Jitendra Mishra, Maheswar Chaudhury, Kushagra Nangia, Beena Devi Agarwal, Pradosh Kumar Sarangi

**Affiliations:** 1 Radiology, Institute of Medical Sciences and SUM Hospital, Bhubaneswar, IND; 2 Orthopedics, Institute of Medical Sciences and SUM Hospital, Bhubaneswar, IND; 3 Radiology, All India Institute of Medical Sciences, Deoghar, Deoghar, IND

**Keywords:** 3t mri, arthroscopy, glenohumeral joint, glenoid labral tears, shoulder

## Abstract

Background

Glenoid labral tears are a common cause of shoulder pain and instability, particularly in young and physically active individuals, and are frequently associated with recurrent dislocations and functional impairment. Accurate diagnosis is essential for appropriate management and prevention of long-term morbidity. While MRI is widely used as a non-invasive modality for evaluating intra-articular shoulder pathologies, arthroscopy remains the gold standard for definitive diagnosis. However, the diagnostic performance of conventional MRI, even at higher field strengths such as 3.0 Tesla, continues to be an area of active investigation. This study aims to assess the correlation between 3-Tesla MRI and arthroscopic findings in the detection of glenoid labral tears and to evaluate the ability of MRI to identify associated variants and coexisting shoulder pathologies.

Methodology

This cross-sectional diagnostic validity study included 50 patients presenting with clinically suspected labral tears. All participants underwent evaluation by a standardized 3.0-Tesla MRI of the shoulder followed by arthroscopy. Tear presence, type, and associated abnormalities were documented. Diagnostic performance parameters, including sensitivity, specificity, positive predictive value (PPV), negative predictive value (NPV), and accuracy, were calculated using arthroscopy as the reference standard. Data were analyzed using SPSS version 20.

Results

MRI detected labral tears in 40 (80%) patients, while arthroscopy identified tears in 43 (86%) patients. The most common tear pattern on both modalities was anteroinferior labral tear in 22 (44%) patients, followed by superior labral anteroposterior lesions. MRI demonstrated sensitivity of 90.7%, specificity of 85.7%, PPV of 97.5%, NPV of 60%, and overall accuracy of 90% for tear detection. Additional pathologies were found in 35 patients, with Hill-Sachs defect in 10 (28.6%), adhesive capsulitis in six (17.1%), and rotator cuff abnormalities in five (14.4%) patients.

Conclusions

MRI shows excellent diagnostic sensitivity and overall accuracy for detecting glenoid labral tears, particularly anteroinferior lesions, and serves as a reliable first-line, non-invasive modality. However, its lower NPV indicates that a normal MRI cannot reliably exclude pathology, affirming the role of arthroscopy in definitive assessment.

## Introduction

The glenohumeral joint, the most mobile joint in the human body, is prone to instability and soft-tissue injuries. Stability is ensured by the joint capsule, glenohumeral ligaments, rotator cuff muscles, and glenoid labrum. The glenoid labrum deepens and increases the surface area of the articulation and serves as the attachment site for the capsuloligamentous complex and the long head of the biceps tendon [1,2]. Shoulder pain and functional impairment are frequent complaints for which cross-sectional imaging is often ordered. Conventional radiography helps detect fractures, osseous abnormalities, and degenerative changes, but is limited by its inability to adequately assess intra-articular soft tissues. Ultrasound of the shoulder is a simple, cheap, and fast non-invasive imaging tool for the detection of rotator cuff and non-rotator cuff abnormalities [3,4].

Ultrasonography allows for dynamic evaluation of superficial structures, such as the rotator cuff and subacromial-subdeltoid bursa. However, ultrasonography is operator-dependent and has limited use in assessing deep intra-articular structures, such as the labrum and the capsuloligamentous complex [2]. Variants of normal anatomy and overlapping imaging appearances can make interpretation challenging, necessitating validation of MRI performance against arthroscopy, optimizing clinical decision-making, and reducing unnecessary invasive procedures. MRI has emerged as the primary non-invasive tool for the comprehensive assessment of glenohumeral joint pathology. The advantages of MRI’s multi-planar capability and high soft-tissue contrast make it an invaluable tool for the assessment of shoulder pathology [5]. The use of 3-Tesla MRI scanners provides better spatial resolution and a higher signal-to-noise ratio, which helps visualize small structures of clinical significance, such as labral anatomy, ligamentous integrity, rotator cuff tendons, and associated osseous pathology, such as Hill-Sachs deformity and glenoid bone loss [6]. MRI is valuable when clinical examination is limited by pain and allows evaluation of regions that may be incompletely visualized arthroscopically, including the subscapularis tendon insertion and the inferior glenohumeral ligament complex [7].

However, despite its advantages, precise evaluation of the glenoid labrum on MRI can be difficult due to partial-volume averaging, the magic-angle effect, and orientation-dependent signal changes, which can affect the identification of minor tears. In addition, there is some variation in normal anatomy, which includes the sublabral recess, sublabral foramen, and Buford complex, which may mimic labral detachment or tears [8]. Thus, an understanding of the imaging spectrum of normal variations and pitfalls that can confuse diagnosis is crucial. Arthroscopy is considered the gold standard for assessing intra-articular abnormalities of the shoulder joint, as it allows visualization of the labrum, biceps tendon, and articular surface [9]. However, arthroscopy is invasive, expensive, and poses risks to patients; hence, assessing the diagnostic accuracy of high-field MRI is critical to optimize patient selection for surgical intervention.

## Materials and methods

This cross-sectional diagnostic study was conducted at the Institute of Medical Sciences and SUM Hospital, Bhubaneswar, Odisha, over two years, from 2023 to 2025. Ethical approval from the Institutional Ethics Committee (Ref. no/IEC/IMS.SH/SOA/2024/945). A convenience sampling method was used to determine the sample size in the study. A total of 50 patients aged 10-80 years who presented with suspected glenoid labral tears of the shoulder, as reported by a referring orthopedician, were included. After obtaining informed consent, patients underwent a 3.0-Tesla MRI, followed by arthroscopy for suspected glenoid labral tears. Respondents were interviewed after providing informed consent and were assured of strict privacy and confidentiality. A structured questionnaire was used for the study with necessary modifications based on the variables used in previous studies. Standard shoulder MRI protocols and arthroscopic evaluation served for comparative analysis. Patients referred for MRI and shoulder arthroscopy who were willing to participate and provide informed consent were included. Non-MRI-compatible metallic implants, the presence of a pacemaker, and patients unwilling to participate were excluded.

The imaging was performed with the patient in the supine position, using a coil and orientation based on the glenoid labrum. MRI parameters were recorded by analyzing the glenoid labrum in the following sequences: T1-weighted, T1-weighted fat-saturated, T2-weighted fat-saturated, and proton density fat-saturated. The images were acquired in axial, coronal oblique, and sagittal oblique planes. The diagnosis was subsequently confirmed based on arthroscopic findings. Arthroscopy can be performed in the beach chair or lateral decubitus position. The lateral decubitus position is often favored for better access to the inferior glenoid and to facilitate an inferior-to-superior capsular shift. Standard portals include the posterior portal for initial diagnostic visualization, the anterosuperior portal for instrumentation and visualization, and the anteroinferior portal, crucial for labral repair. The primary outcome of the current study was the detection and localization of a labral tear, and the secondary outcome was the detection of additional shoulder joint pathologies and their correlation with arthroscopy for diagnostic accuracy.

Statistical analysis

Data from all participants were entered into MS Excel (Microsoft Corp., Redmond, WA, USA) and analyzed using SPSS version 20 (IBM Corp., Armonk, NY, USA). In the present study, analysis was performed per shoulder. Categorical variables were expressed as frequencies and percentages, and continuous variables as mean ± SD. Diagnostic performance of MRI was evaluated against arthroscopy using sensitivity, specificity, positive predictive value (PPV), negative predictive value (NPV), and accuracy. A p-value <0.05 was considered statistically significant. Additionally, the collected data were analyzed for agreement between MRI and arthroscopic findings using Cohen’s (simple) kappa statistics.

## Results

A total of 50 patients with clinically suspected glenoid labral tears were included. The cohort consisted of 35 (70%) men and 15 (30%) women, with a mean age of 42.0 years (range = 18-80 years) (Table 1).

**Table 1:** Distribution of gender.

Gender	Frequency	Percentage
Male	35	70
Female	15	30
Total	50	100

The most common age groups were 31-40 years and 51-60 years. On MRI, labral tears were identified in 40 of 50 (80%) patients, whereas arthroscopy demonstrated tears in 43 (86%) patients (Table 2).

**Table 2:** Distribution of age.

Age group (years)	Frequency	Percent
11–20	5	10
21–30	10	20
31–40	11	22
41–50	6	12
51–60	11	22
61–70	6	12
71–80	1	2
Total	50	100

**Figure 1:**
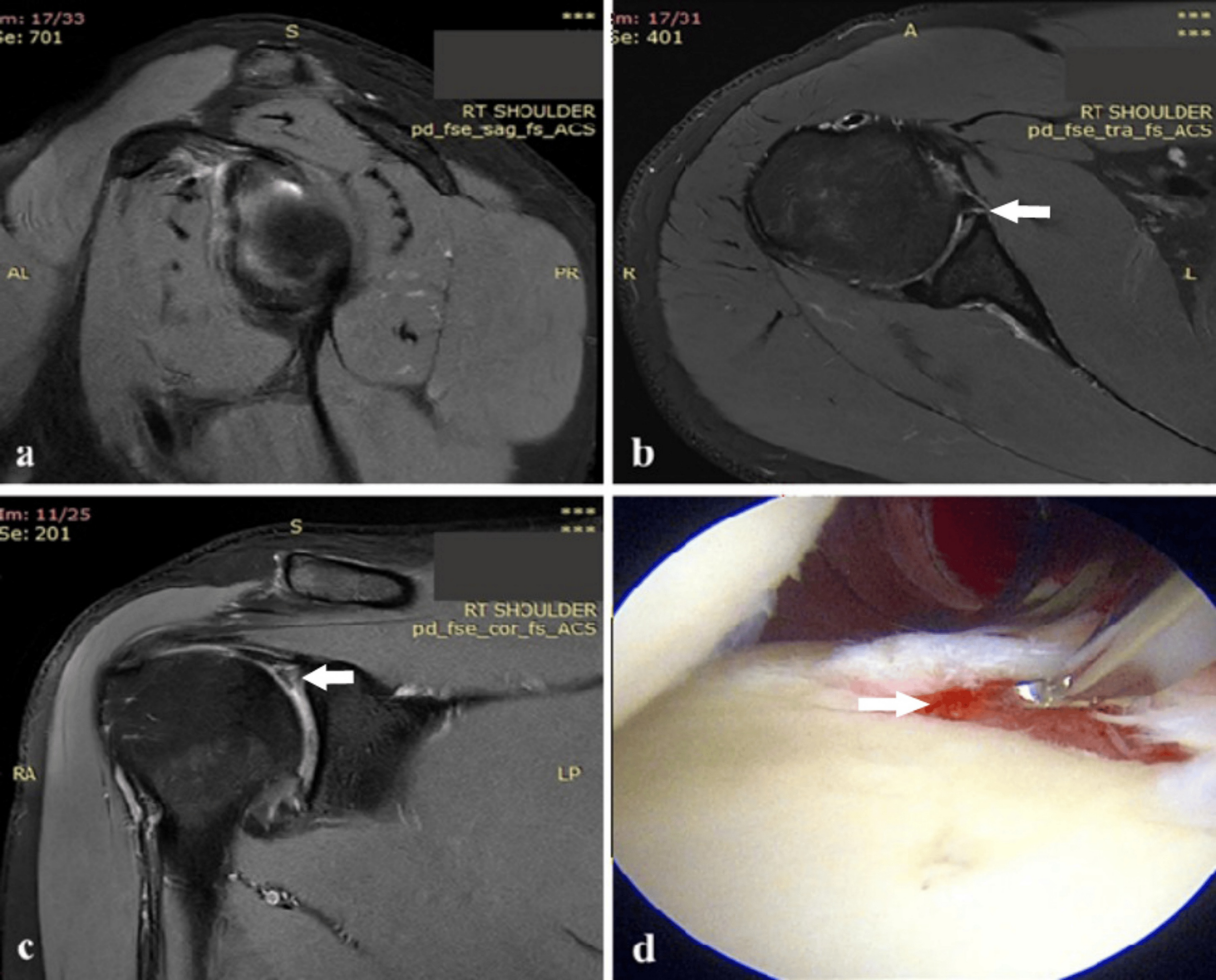
A 26-year-old male with shoulder pain and instability. (a, b) Sagittal and axial PDFS images show a hyperintense cleft in the anterior glenoid labrum extending from the 12 to 6 o’clock position. (c) The coronal PDFS image demonstrates a hyperintense signal in the superior labrum. (d) Arthroscopy confirms the labral tear, followed by arthroscopic repair. PDFS: proton density fat-saturated

The most common tear type on MRI was anteroinferior (44%), followed by anterosuperior (10%), posterosuperior (8%), superior labral anteroposterior (SLAP) tears (8%), and posteroinferior tears (6%) (Figure 1). Less common lesions included glenolabral articular disruption and posterior labrocapsular periosteal sleeve avulsion lesions (2% each). Overall, anterior labral involvement accounted for approximately 54% of cases, posterior involvement for 14%, and superior labral involvement, including SLAP lesions, for 26%. Inferior labral involvement was observed in 50% of patients. The most common clinical symptoms were shoulder pain in 43 (86%) patients, pain during activities in 32 (64%) patients, and instability in 22 (44%) patients (Table 3).

**Table 3:** Clinical symptoms of the study.

Clinical findings	Frequency	Percentage
Shoulder pain	43	86
Clicking popping/grinding sensation	20	40
Instability	22	44
Decreased range of motion or stiffness	12	24
Pain during activities	32	64

**Figure 2:**
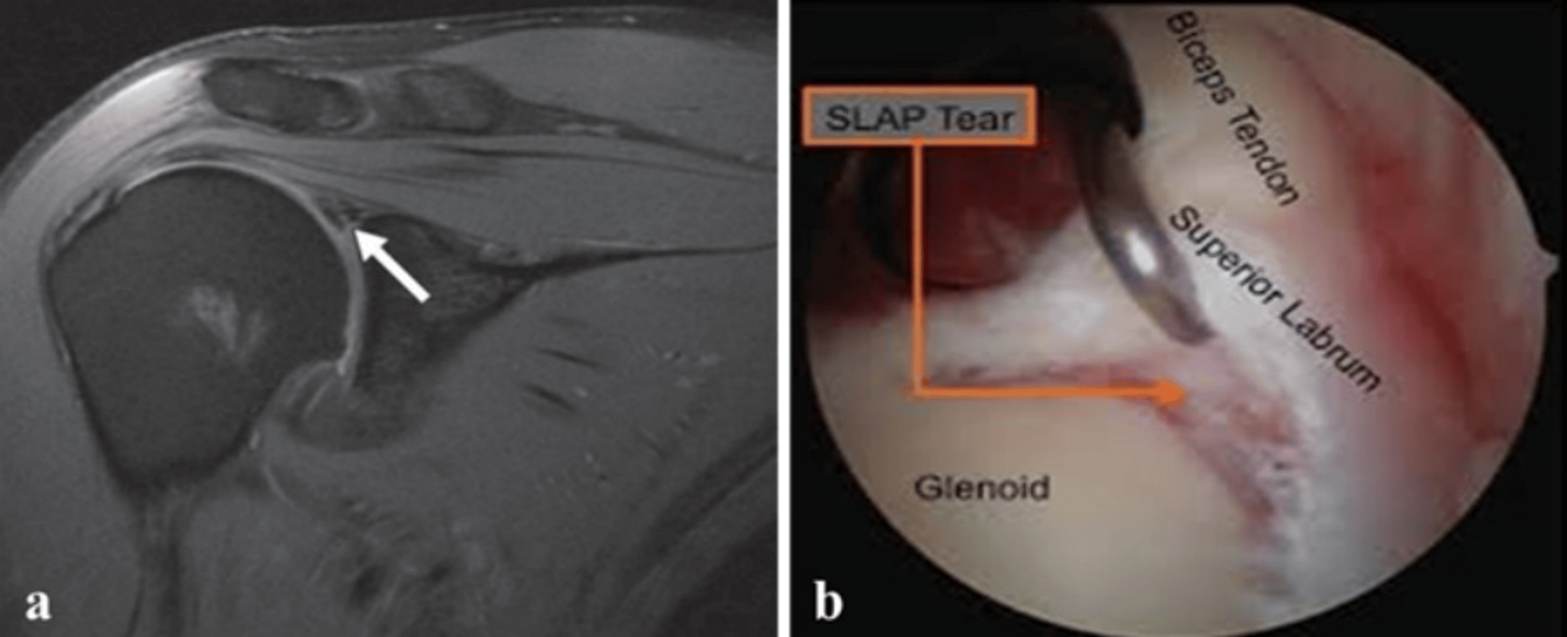
A 43-year-old male presenting with clicking and popping sensations during shoulder movement. (a) The coronal PDFS image shows a hyperintense signal extending into the superior labrum (arrows). (b) Arthroscopy confirms a SLAP tear. PDFS: proton density fat-saturated; SLAP: superior labral anteroposterior

Arthroscopic findings demonstrated a similar distribution, with anteroinferior tears being the most common (44%), followed by SLAP tears (12%), anterosuperior tears (10%), and posterior tears (8% each for posterosuperior and posteroinferior) (Figure 2). No tear was identified in 14% of patients on arthroscopy. MRI demonstrated sensitivity of 90.7%, specificity of 85.7%, PPV of 97.5%, NPV of 60%, and overall diagnostic accuracy of 90% (Table 4).

**Table 4:** Diagnostic performance of MRI in the evaluation of glenoid labral tears (arthroscopy as the reference standard). Exact 95% confidence intervals for sensitivity, specificity, predictive values, and accuracy were calculated using the Clopper–Pearson method based on the binomial distribution.

Parameter	Value (%)	95% confidence interval (%)
Sensitivity	90.7	77.9–97.4
Specificity	85.7	42.1–99.6
Positive predictive value	97.5	86.4–99.6
Negative predictive value	60.0	35.6–80.0
Accuracy	90.0	78.2–96.7

MRI detected labral tears with a Cohen’s unweighted kappa of 0.65 (95% confidence interval = 0.36-0.94), indicating substantial agreement with arthroscopy (Table 5). Associated abnormalities were present in a substantial proportion of patients, most commonly Hill-Sachs lesions (45.8%), including engaging (28.6%) and non-engaging (17.2%) types. Other findings included adhesive capsulitis (17.1%), rotator cuff tears (11.5%), inferior glenohumeral ligament thickening (5.7%), and paralabral cysts (5.7%).

**Table 5:** Contingency table comparing MRI results with arthroscopic findings for the detection of glenoid labral tears.

	Arthroscopy positive	Arthroscopy negative	Total
MRI positive	39 (true-positive)	1 (false-positive)	40
MRI negative	4 (false-negative)	6 (true-negative)	10

## Discussion

In the present study, the majority of patients were males (70%), similar to findings in previous research by Ernat et al. and Cronin et al., where a higher occurrence of shoulder trauma was noted in males [4,6]. The average patient age was 42.02 years, with only 30% less than 30 years old, meaning that this group of patients is mostly older adults who have labral lesions. Cronin et al. found that the mean age was significantly lower (24.7 ± 9.0 years) due to the inclusion of all cases of shoulder instability; the current study was limited only to labral tears [6]. Based on tear patterns observed through MRI, the most frequent type was anteroinferior tears (44%), and anterior labral tears accounted for 54% of cases. The high incidence of anteroinferior tears in the present study is similar to that observed in earlier research conducted by Waldt et al. and Magee et al., both of whom noted a similar pattern [3,5], thereby emphasizing the well-known correlation between anterior instability and labral tear. According to Chandnani et al., there was a relatively higher incidence of anterior and superior labral tears, but the study used classification criteria based on overall regional involvement rather than proportional tear incidence [1]. Minor discrepancies can be attributed to these factors. In the current study, 80% of tears were confirmed by MRI and 86% by arthroscopy, suggesting MRI underestimates tear prevalence. This trend was also observed in prior literature, where Chandnani et al. reported prevalence rates of about 93% with MRI and 96% with MR arthrography [1].

The diagnostic efficiency of MRI in the present study was high, with sensitivity, specificity, and overall accuracy at 90.7%, 85.7%, and 90%, respectively. A very high PPV of 97.5% confirms MRI’s high reliability in diagnosing labral tears. However, the lower NPV of 60% suggests that a negative MRI may not reliably rule out a labral tear, and thus, arthroscopic evaluation is still required in those patients. This finding is consistent with previous studies by Bhatnagar et al. and Smith et al. [10,11]. Furthermore, the markedly low sensitivity for SLAP lesions reported by de Almeida et al. further supports the potential for underdiagnosis of these lesions on MRI [12].

SLAP injuries were seen in 8% using MRI and 12% using arthroscopy. This suggests that SLAP injuries may be underdiagnosed using MRI secondary to their subtle appearance and difficulty differentiating from anatomical variation. Identification using conventional MRI is additionally hampered by the curved anatomy of the superior labrum and the lack of joint distention. The difficulty in identifying SLAP injuries on MRI stems from their nature. SLAP injuries involve detachment at the biceps tendon attachment site; their diagnosis is challenging owing to their morphological diversity and subtle appearance [13]. Among rotator cuff injuries, the majority of supraspinatus tendon injuries were full-thickness tears (80%). This was consistent with findings from a systematic review by Lenza et al., in which MRI was noted to have excellent diagnostic accuracy for detecting full-thickness tears, while its sensitivity for partial-thickness tears was relatively poor [14].

Several conditions could account for differences between MRI and arthroscopy results, including normal intralabral signal changes, variation in labral morphology, and the absence of joint effusion, making it difficult to detect minute tears [15]. McCauley et al. observed that 27% of their patients with normal shoulders showed an intralabral signal extending through the labrum. Furthermore, anatomical abnormalities such as the sublabral recess, sublabral foramen, and Buford complex could be confused with tears of the labrum, especially in the anterosuperior quadrant [8]. The diagnostic evaluation of the anterosuperior portion of the glenoid labrum remains challenging and requires a thorough understanding of shoulder anatomy. As a result, SLAP injuries may be confused with normal anatomical variations of the labrum, and vice versa [8,16]. The analysis of agreement using Cohen’s kappa test revealed a kappa coefficient of 0.65, indicating significant agreement between MRI and arthroscopy. This supports MRI’s reliability as a noninvasive modality for evaluating glenoid labral pathology.

Limitations

Limited sample size, technical factors, including partial-volume averaging, magic-angle artifact, and cartilage undercutting, may further contribute to diagnostic challenges. MRI may miss subtle labral tears or incorrectly identify normal anatomical variants as tears. While arthroscopy is considered the gold standard, this procedure is limited and has blind spots that may miss extra-articular pathology.

## Conclusions

The 3-Tesla MRI is an efficient, non-invasive method for evaluating shoulder labral tears. Arthroscopy remains the gold standard for diagnosing shoulder joint pathologies, but MRI can be a helpful non-invasive method for assessing glenohumeral joint injuries. MRI demonstrated satisfactory diagnostic performance, with high sensitivity, specificity, and accuracy. An anteroinferior labral tear was the most common tear detected by both methods used in the study, underscoring MRI’s ability to accurately diagnose clinically significant patterns of shoulder joint instability. MRI helped detect other concomitant pathologies, namely, Hill-Sachs lesions, rotator cuff tears, paralabral cysts, and capsuloligamentous injury. Hence, 3.0-Tesla MRI can be considered a dependable first-line diagnostic tool, with arthroscopy reserved for therapeutic intervention or ambiguous cases.

## Figures and Tables

**Figure 3:**
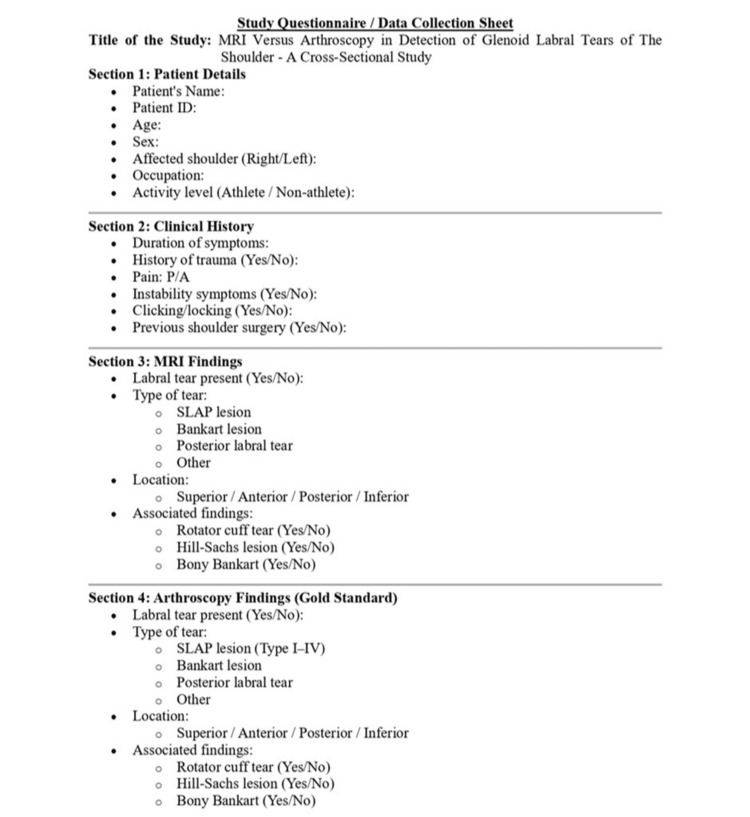
Structured questionnaire used in this study.
